# Implementing agricultural phosphorus science and management to combat eutrophication

**DOI:** 10.1007/s13280-015-0631-2

**Published:** 2015-02-15

**Authors:** Peter J. A. Kleinman, Andrew N. Sharpley, Paul J. A. Withers, Lars Bergström, Laura T. Johnson, Donnacha G. Doody

**Affiliations:** 1 USDA-ARS Pasture Systems and Watershed Management Unit, University Park, PA 16802 USA; 2 Department of Crop, Soil and Environmental Sciences, University of Arkansas, Fayetteville, AR 72701 USA; 3 School of Environment, Natural Resources and Geography, Bangor University, Bangor, LL57 2DG UK; 4 Department of Soil and Environment, Swedish University of Agricultural Sciences, P.O. Box 7014, 75007 Uppsala, Sweden; 5 National Center for Water Quality Research, Heidelberg University, Tiffin, OH 44883 USA; 6 Agri-food and Bioscience Institute, Newforge Lane, Belfast, 8T9 5PX UK

**Keywords:** Eutrophication, Mitigation measures, No till, Phosphorus management, Tile drainage

## Abstract

Experience with implementing agricultural phosphorus (P) strategies highlights successes and uncertainty over outcomes. We examine case studies from the USA, UK, and Sweden under a gradient of voluntary, litigated, and regulatory settings. In the USA, voluntary strategies are complicated by competing objectives between soil conservation and dissolved P mitigation. In litigated watersheds, mandated manure export has not wrought dire consequences on poultry farms, but has adversely affected beef producers who fertilize pastures with manure. In the UK, regulatory and voluntary approaches are improving farmer awareness, but require a comprehensive consideration of P management options to achieve downstream reductions. In Sweden, widespread subsidies sometime hinder serious assessment of program effectiveness. In all cases, absence of local data can undermine recommendations from models and outside experts. Effective action requires iterative application of existing knowledge of P fate and transport, coupled with unabashed description and demonstration of tradeoffs to local stakeholders.

## Introduction

The challenges of mitigating diffuse phosphorus (P) pollution are manifold, but no more complex than in the arena of implementing P-based management in agricultural watersheds. Phosphorus-based practices and the strategies that guide the implementation of these practices, once considered novel in the 1990s, have now been tried across North America and Europe, providing an ever-growing wealth of experience. These experiences highlight the iterative interaction of applied science and social experimentation that comes from trying to modify fundamental aspects of our food production and conservation systems.

The science and practice of implementing nutrient management strategies are often disconnected in watershed management, even though both are key to the success or failure of watershed remediation. It is clear that the science of understanding how P management affects water quality and the implementation of management practices via voluntary and coercive means are mutually dependent. Science provides justifications and narratives to underscore or drive implementation processes. Implementation guides or constrains the range of options considered by applied science. At its best, this tautology, or feedback loop, represents the process of adaptive management. However, one must recognize that this reinforcing process invariably absorbs assumptions and biases that are unrecognized by those involved.

Both “sacred cows” and their converse, “sacrificial lambs” are regularly encountered in the science and implementation of P management. Sacred cows can be found in the assumptions that certain environmental processes and management practices are so established that they are left unquestioned or perennially advocated. Sacrificial lambs, on the other hand, may occur as phenomena that are underestimated in their importance to the fate and transport of P or as practices that are readily condemned without due assessment. Recognizing the presence of these biases is required for objective and sustainable management solutions.

Even without sacred cows and sacrificial lambs, the challenges facing P management can vary dramatically, dependent upon local production systems, physiography, history, culture and politics, and, of course, economics and policies. Although there is a long history of watershed programs grappling with P management, efforts to confront diffuse sources of P, i.e., non-point sources of P, are often placed on the periphery, or margin, of other agricultural and conservation initiatives. When traditional conservation programs are insufficient to control diffuse P losses, watershed P problems are often described as novel (e.g., dissolved P loadings via tile drains) or unforeseen (legacy sources of P). In part this marginalization reflects the secondary nature of P as a plant nutrient, when compared with nitrogen (N). However, as we will seek to illustrate, the ubiquity of P sources and the degree of P management required for successful watershed outcomes are often underestimated (willfully or inadvertently).

To understand successes and challenges in P-based management, we review case studies from North America and Europe, delving into the challenges and opportunities associated with voluntary and regulated approaches to implementation. In general, North American experiences have emphasized voluntary adoption, whereas European experiences have followed regulations. Even so, all shades of coercion can be found on both continents. Beginning with the recent, highly publicized case of Lake Erie, whose resurgent water quality problems point to the vexing nature of P-based management, we review case studies with obvious, and less obvious, sacred cows and sacrificial lambs, highlighting uncertainty, successes, and factors affecting strategic and tactical outcomes.

## Voluntary efforts in Western Lake Erie, USA

**Fig. 1:**
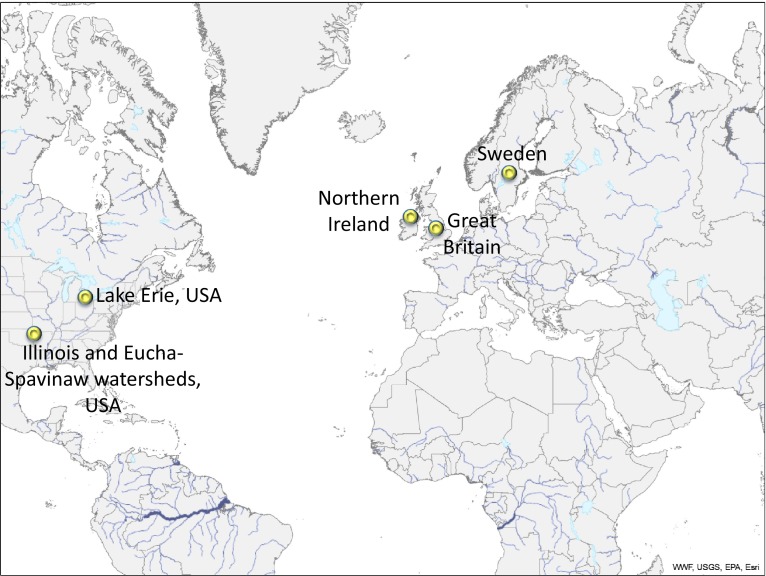
Map of case study locations in the USA, UK, and Sweden

Agricultural P management has re-emerged as a priority concern in Lake Erie (Fig. 1), one of the Great Lakes bordering the USA and Canada and the site of historical successes in P mitigation. In 2014, prevailing winds directed a cyanobacterial bloom from Western Lake Erie into the drinking water intake for the City of Toledo, causing a spike in the toxin microcystin that overwhelmed the treatment facility. Toledo had to halt water supply to 400 000 users, prompting calls for strict regulations on agricultural P, which has so far been subject to voluntary management (White 2014).

Today’s dissolved P concerns in Western Lake Erie contrast with the historical success of point and non-point source P control programs in helping to lower P loads to the lake (Richards et al. 2009). From 1975 to 1995, loads of total P from the two largest watershed inputs, the Maumee and Sandusky Rivers, declined by 75 % and loads of dissolved P declined by 50 % (Sharpley et al. 2012). Agricultural conservation efforts targeting highly erodible lands contributed to substantial reductions in sediment and particulate P in runoff and stream flow. Nutrient management planning, particularly the prescription of fertilizer P rates based upon soil test P levels and crop needs, resulted in overall reductions of P applied as fertilizer of more than 30 % and as manure of 25 % (Baker and Richards 2002; Richards et al. 2002).

**Fig. 2:**
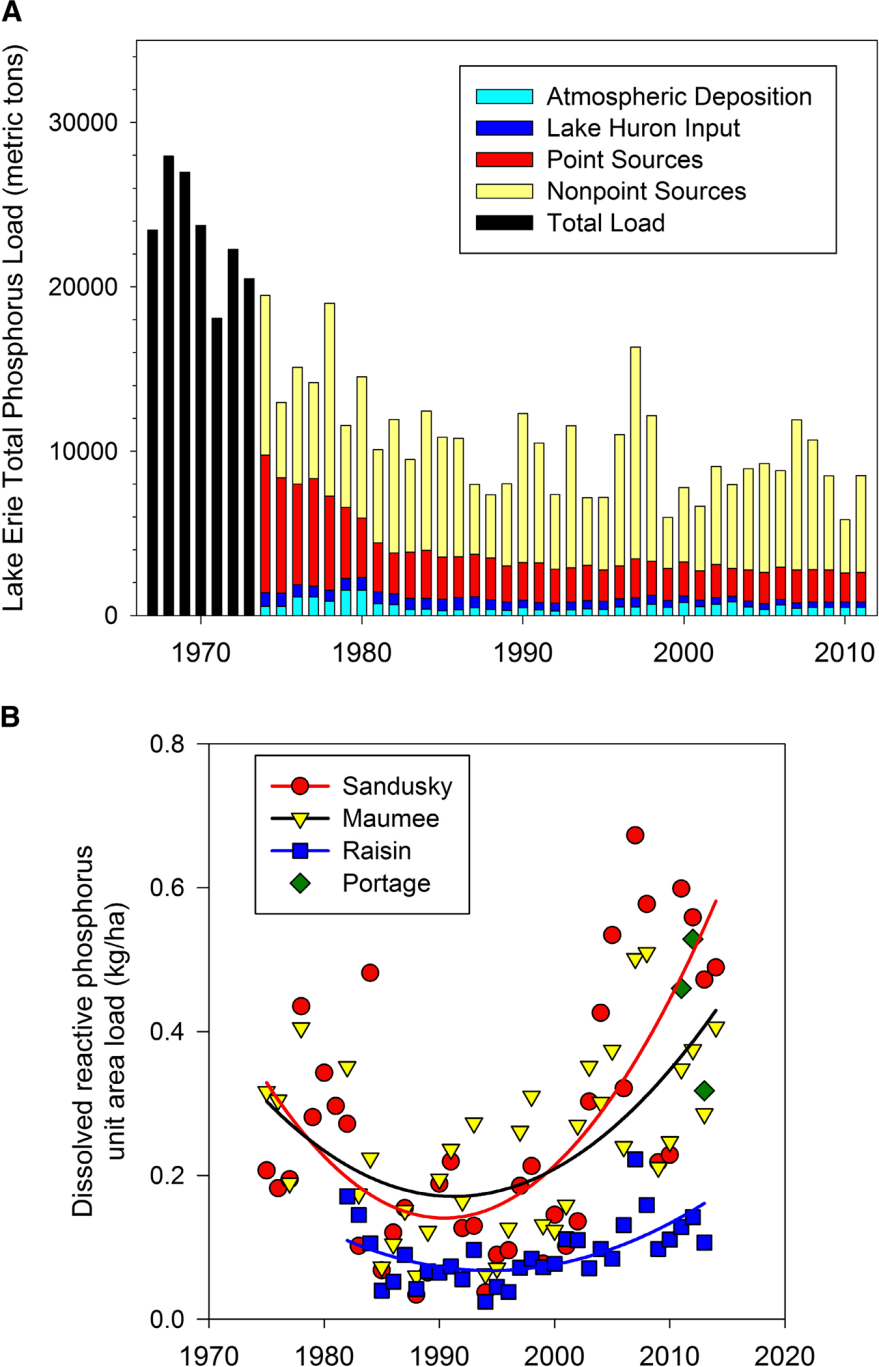
Long-term trends in **a** total P loads and **b** dissolved P loads from Western Lake Erie watersheds to Lake Erie. Data are adapted from Dolan and Chopra (2012) and International Joint Commission (2014)

On the heels of these tremendous improvements in Lake Erie water quality, an uptick in dissolved P loads occurred in the mid-1990s, despite the persistence of historically low total P loads. Since 1995, dissolved P loads from Western Lake Erie Watersheds have increased (Fig. 2; Baker et al. 2014), triggering harmful algal blooms (Stumpf et al. 2012; Michalak et al. 2013). Identifying and controlling agricultural sources of dissolved P to Western Lake Erie has been difficult. Even climate change is a factor. Recent shifts in annual rainfall distribution have resulted in more intense rains in spring months during the 5 years, compared with the previous 10 years (Joosse and Baker, 2011; Smith et al. 2014), increasing the potential for P runoff at a vulnerable time for agriculture and a sensitive time for lake response (Chaffin et al. 2011). Furthermore, loads to Western Lake Erie from agricultural fields are low, on the order of 1–2 kg P ha^−1^ year^−1^ (Smith et al. 2014), complicating the identification of culprits and making room for competing narratives.

### No-till

Nowhere is the debate over sources of agricultural dissolved P to Western Lake Erie more polarized than in the complex realm of no-till, or reduced tillage, and fertilizer management. During the 1980s and 1990s, various forms of no-till were rapidly adopted throughout the region, with their peak expansion in the mid-1990s coincident with the first dissolved P increases in Western Lake Erie (Richards et al. 2002; Sharpley et al. 2012; Baker et al. 2014). No-till adoption has been voluntary, driven by benefits to farm profitability through fewer field operations (saving in time, labor, and energy) and aggressive promotion by industry and conservation agencies.

A dogmatic “never till” sentiment exists within segments of the conservation community, one that is buoyed by the fact that very little permanent no-till exists in the region—most farmers employ some form of tillage, increasingly vertical tillage, at some point during their crop rotation. It should not be surprising, therefore, that farm and conservation groups alike have been skeptical of claims that no-till exacerbates dissolved P losses. This skepticism has been aggravated by accusations from groups outside the farming community that no-till is at the heart of agriculture’s dissolved P problem (e.g., a press release by a national environmental organization that no-till was the primary cause of Lake Erie’s P loadings). The effect of such accusations has been entrenchment of alternative perspectives and the simultaneous transformation of no-till into both a sacred cow and sacrificial lamb.

No-till, and even reduced tillage, is associated with well-documented trade-offs when it comes to diffuse P pollution, particularly in cropping systems where fertilizers or manures are broadcast onto the soil surface, as opposed to banded at time of application, injected or otherwise incorporated (e.g., Sharpley and Smith 1994; Tiessen et al. 2009). While particulate P losses in runoff are largely curtailed with no-till, dissolved P losses can increase with no-till. Indeed, decreases in particulate P concentrations along with suspended sediments have been documented in the Western Lake Erie Watersheds (Richards et al. 2008).

No-till can exacerbate the direct transfer of broadcast fertilizer or manure P to runoff (surface and subsurface). Referred to as “incidental transfer” (Preedy et al. 2001; Withers et al. 2003) or “wash-off” (Buda et al. 2013), this form of dissolved P transfer represents an acute risk (i.e., punctuated in time) that is modified by the rate, timing, placement and form of P application. One further concern with no-till in the Lake Erie region is the fact that P fertilizer is typically broadcast in the fall and winter, even to frozen soils, and often at rates to meet crop P requirements for several years. Although there exists debate over some factors affecting dissolved P wash-off (e.g., timing), for the most part better management of applied P sources lends itself to educational programs, such as the “4Rs” of nutrient stewardship (Henry 2014), promoted by the fertilizer industry’s International Plant Nutrition Institute and USDA’s Natural Resources Conservation Service, among others (International Fertilizer Association 2009; International Plant Nutrition Institute 2014).

Also well recognized as a key set of P transfer processes is the accumulation of P in surface soil with repeated application of fertilizer or accumulation of plant residue. This phenomenon is more gradual than the wash-off phenomenon, but, with time, accumulated P elevates background concentrations of dissolved P in runoff waters (surface and subsurface) through metastable processes controlling P sorption and desorption (Kleinman et al. 2011). Vertical stratification of P in soil can be underestimated, even overlooked, through traditional agronomic soil sampling, which mixes the veneer of P-saturated soil at the surface (<2-cm) with sub-soil (typically up to 15-cm depth) that buffers surface P concentrations (Sharpley 2003). Research at Heidelberg University in Western Lake Erie Watersheds has documented significant vertical stratification of P in agricultural soils, despite the fact that soils in continuous no-till accounted for only 8 % of fields sampled and rotational no-till accounted for 59 % of fields sampled (Heidelberg University 2010). In these surveys, mean Mehlich-3 soil P concentrations averaged 60 mg kg^−1^ in surface samples (0–2.5 cm) and 36 mg kg^−1^ in traditional agronomic samples (0–12 cm).

Vertical nutrient stratification can be reduced through subsurface placement of fertilizer, such as with banding, particularly deep banding (>15 cm), which also provides yield benefits (Mallarino et al. 1999; Mallarino and Borges 2005, 2006). An even more effective means of addressing vertical P stratification in soils is through tillage, particularly tillage that inverts (e.g., moldboard) and mixes (e.g., vertical) to ensure dilution of surface P and contact of highly P saturated soil particles with particles that have a high P buffering potential (e.g., Sharpley 2003). Tillage can also disrupt leaching of applied P (e.g., Kleinman et al. 2009). Recommendations for even infrequent tillage of highly vertically stratified sites, however, have been met with categorical rejection from the strongest no-till advocates, even when suggested as an infrequent remedy. Many in the conservation community have long histories of promoting soil conservation practices, such as “park the plow.” Condoning tillage after decades of soil conservation outreach is seen as anathema to prudent conservation messaging.

### Tile drainage

Artificial drainage—tile drainage but also surface inlets and drainage ditches—has long served as the foundation for successful crop production in the Lake Erie region. In recent years, there has been a substantial, but poorly quantified, intensification of artificial drainage. As farmers take advantage of tax credits related to infrastructure improvement, investing profits in drainage, there has been an increase in the purchase of tiling plows that allow farmers to install tile lines themselves, rather than to contract out the work, adding incentive to recoup purchase costs by installing more tile drains. So profitable is the investment of artificial drainage and so important is drainage to agronomic improvement that few in the agricultural community have been willing to confront this sacred cow.

The potential for substantial P loss via tile drains is met with skepticism and even denial in conservation and farm communities alike (Egan 2014). This perception belies a long history of science on P loss from tile drains, largely in other regions (King et al. 2014a). One argument repeated by those skeptical that tile drainage can exacerbate agricultural P losses is that drainage should decrease surface runoff, the dominant pathway of P transport in most systems. Tile drainage, proponents posit, lowers antecedent soil moisture and improves infiltration, decreasing P loss potential. Standing in contrast is the argument that tile drainage creates hydrologic connectivity to areas that would otherwise be disconnected from the preferential flow pathways required for P transfer. Both arguments are currently difficult to categorically prove or disprove, given the range of drainage configurations that exist and the poor ability of computational models to simulate P loss in tile drainage (Radcliffe et al. 2014).

So different is subsurface P transport from other, well-understood drainage concerns (e.g., N transport), that empirical observations remain the gold standard in this highly polarized environment. Considerable federal and state funding has recently gone into edge-of-field monitoring in the Lake Erie region, confirming the role of artificial drainage as a major pathway for dissolved and particulate P forms alike (King et al. 2014b, Smith et al. 2014). While local empirical data are clearly important to assigning responsibility and convincing local skeptics, the effect of placing the onus of confirmation on local data sources (rather than well established science from elsewhere) has been to delay critical discussions over the installation of new drainage. An unstated programmatic concern has been that too much emphasis on curtailing tile drainage will alienate the agricultural community from voluntary water quality efforts. Instead, there has been a more politically palatable promotion of practices that can be applied after new drainage is installed (e.g., drainage control structures). These practices warrant emphasis but are often expensive and difficult to manage according to best practice. Until objective discussion can be established around tile drainage, it will remain a sacred cow to the agricultural community and a sacrificial lamb to the environmental community.

### Common ground: Improving planning and education

Because P mitigation strategies in Western Lake Erie must prevent dissolved P losses (Ohio Lake Erie Task Force 2013), significant efforts are underway to improve fertilizer and manure management planning and education. A new P Index is being developed in Ohio, validated with edge-of-field monitoring, to better address dissolved P losses and tile drainage (the current Ohio P Risk Index primarily addresses particulate P loss in surface runoff). The fertilizer industry and conservation communities are strongly promoting a “4R certification program” that provides voluntary certification for agricultural retailers to help prevent poor practices and educate growers (http://4rcertified.org/). Educational regulations are appearing; Ohio now requires fertilizer applicators on farms over 20 hectares to attend an educational session on fertilizer application. These efforts have broad support, tying local empirical data and sound agronomic practice to address part of Lake Erie’s water quality woes.

## P-based litigation in the Illinois and Eucha–Spavinaw watersheds, USA

While many of the P management concerns for agriculture in the Western Lake Erie region are directed toward crop production, across the USA, intensive livestock production has received the majority of the attention related to diffuse P losses from agriculture (U.S. Environmental Protection Agency 2013). The Illinois River and Eucha-Spavinaw watersheds span the states of Arkansas and Oklahoma and have been the focus of litigation aimed at a burgeoning poultry industry and expanding urbanization (Fig. 1). A rapid, five-fold increase in the human population in Northwest Arkansas over the last 20 years has coincided with the expansion of confined poultry broiler operations, which now produce over 2000 million birds annually, nearly 25 % of the total broiler production in the USA. Under the U.S. Clean Water Act of 1972, the U.S. Environmental Protection Agency has ruled that upstream watershed users are responsible for downstream water quality. As a result, in 2001, the City of Tulsa, Oklahoma and in 2004 the Attorney General of Oklahoma filed law suits to mitigate the accelerated eutrophication of municipal water supplies, Eucha-Spavinaw reservoirs, and Lake Tahlequah.

As expected, the litigation had an initial effect of polarizing communities—rural and urban, Arkansas and Oklahoma—with competing narratives reflecting an array of long-standing biases and perspectives that had little to do with the sources and causes of watershed P loadings (Sharpley et al. 2012). As with Lake Erie, dissolved P was highlighted as a primary concern, making traditional soil conservation practices inadequate for mitigation, particularly as most productive agricultural lands were pastures (Sharpley et al. 2007). To move litigation toward settlement, the presiding Judge required that site assessment must be carried out to prevent application of poultry litter to fields in the watershed that were at high risk of P loss in runoff. The Judge mandated the use of a P Index that was developed specifically for the unique pastures, topography, and climate of the area (DeLaune et al. 2006).

A competing set of site assessment tools was proposed by experts from Arkansas, which accounts for the majority of the land area of the watersheds, and Oklahoma. Ultimately, Arkansas’ site assessment tool was adopted by the court. During this process, the Judge imposed various requirements on site assessment that, while ostensibly representing compromise, actually complicating standard procedures. Most notably, the Judge imposed a soil P ceiling for litter application, preventing application to soils with Mehlich-3 P > 300 mg kg^−1^ (DeLaune et al. 2006). In 2013, as part of a court settlement agreement, this threshold was made much more restrictive: now P cannot be applied to soils with Mehlich-3 P > 150 mg kg^−1^.

**Fig. 3:**
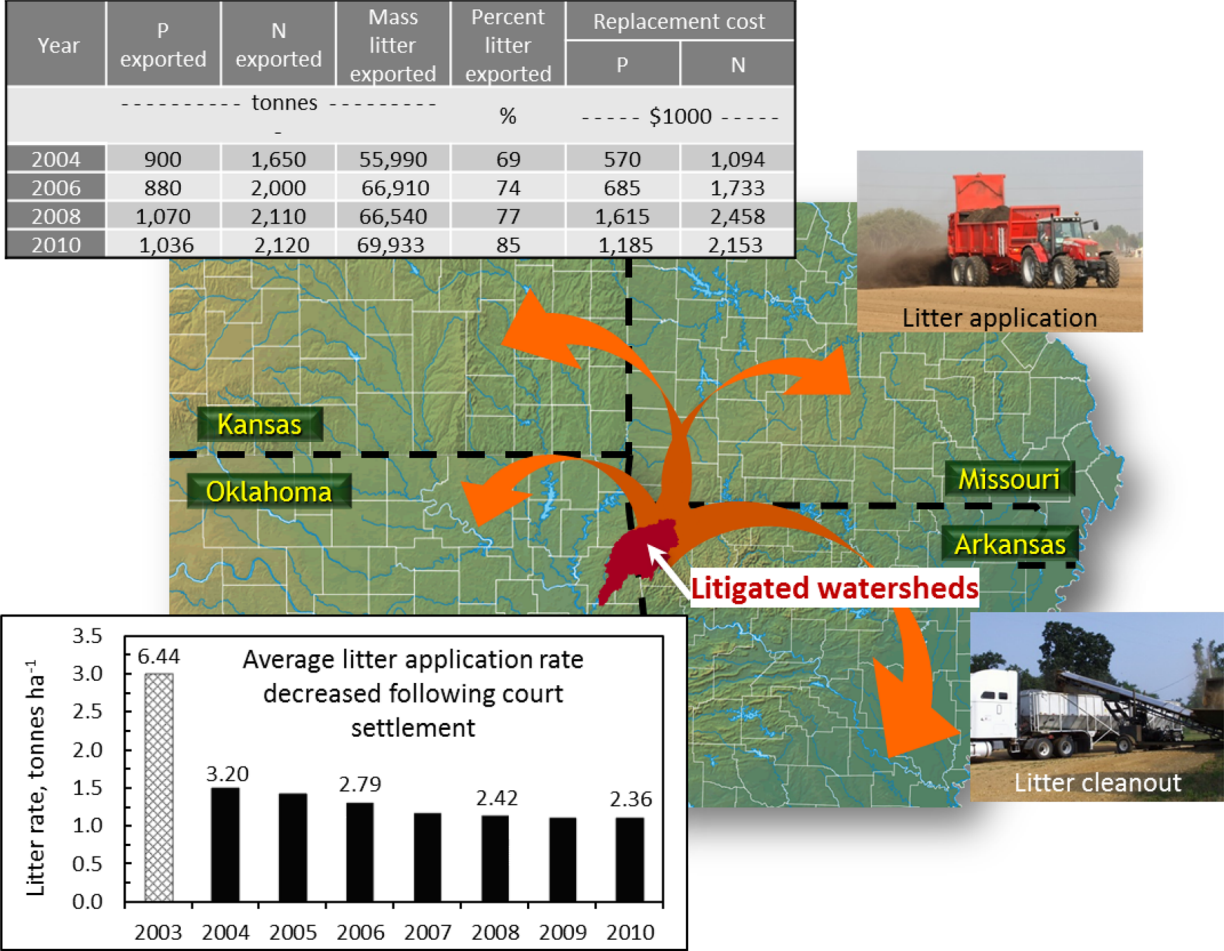
The Illinois River and Eucha-Spavinaw poultry litter export program has been successful in transporting litter from litigated watersheds to agricultural lands in Kansas, Missouri, and Oklahoma as a substitute for commercial fertilizer

The litigation-derived P management standards have led to a marked decrease in the rates of litter applied in the Eucha-Spavinaw and Illinois River watersheds (from ~100 to 40 kg P ha^−1^ year^−1^) (Sharpley et al. 2012). Additionally, the Judge required at least 33 % of all litter produced in the Eucha-Spavinaw Watershed be exported, leading to the development of one of the only viable manure export programs in the USA. The success of the manure export program stems from the nature of the manure (dry poultry litter, which lends itself to transport due to low moisture content, high nutrient content, and a positive image as an organic fertilizer amendment), and from repeated adaptation of export tactics based upon the court-mandated requirement for balancing P application on agricultural soils (Fig. 3). The export of poultry litter out of northwest Arkansas and northeast Oklahoma to non-litigated watershed amounts to over 85 % of the litter produced in the Eucha-Spavinaw Watershed (Fig. 3; Herron et al. 2012; Sharpley et al. 2012). In the Illinois River Watershed, where the lawsuit has not reached a settlement phase, it is more difficult to determine amounts exported, but they are estimated at 100 000 tons, or roughly 37 % of litter produced in the watershed (approximately 275 000 tons in 2010; Herron et al. 2012). Nitrogen exported in litter from the Eucha-Spavinaw Watershed increased from a value of $1 094 000 USD in 2003 to $2 153 000 USD in 2010 (Fig. 3). Assuming that no P would be needed on the pastures when litter is exported, this equates to a significant cost to local beef farmers to buy commercial fertilizer N to maintain pasture productivity.

Key lessons learned include the imperative that export programs are tied to the logistics of importation so that litter can substitute with commercial fertilizer. In the Arkansas watersheds, key ties have been made between the fertilizer industry, particularly distributors, and the livestock industry so that mutual goals are achieved. Increasingly, farmers in the USA derive their nutrient recommendations from private sources, making them a key partner. These ties need to extend to areas where manure is exported, a process that is recognized in Arkansas but ongoing. In Lake Erie, the tie to the fertilizer distributors has been made through voluntary certification to promote environmental stewardship within the industry and, potentially, to provide a marketing edge for certified nutrient management planners.

Despite initial concerns that the restrictions placed by the court case would force poultry growers out of the litigated watersheds, poultry farmers have adapted to the P-based regulations, in part through subsidies supporting manure export. As a result, this case study represents an important example of the potential for farmers to overcome the impacts of mandated manure application restrictions. However, beef farmers (primarily cow-calf) in the area have suffered from the loss of a cheap and plentiful source of N in poultry litter that has enabled profitable cattle production on local pastures. The export of poultry litter under the litigated settlement has produced a slow decline in beef herd size and pasture productivity, coupled with an increased potential for erosion due to worsening pasture conditions with declining fertility. In order to maintain the economic viability of all farming enterprises, not just the poultry farms, it has become clear that the nutrient management planning process must go beyond addressing poultry litter application rates and environmental risk and include educational efforts to help farmers develop sustainable whole-farm operations.

**Fig. 4:**
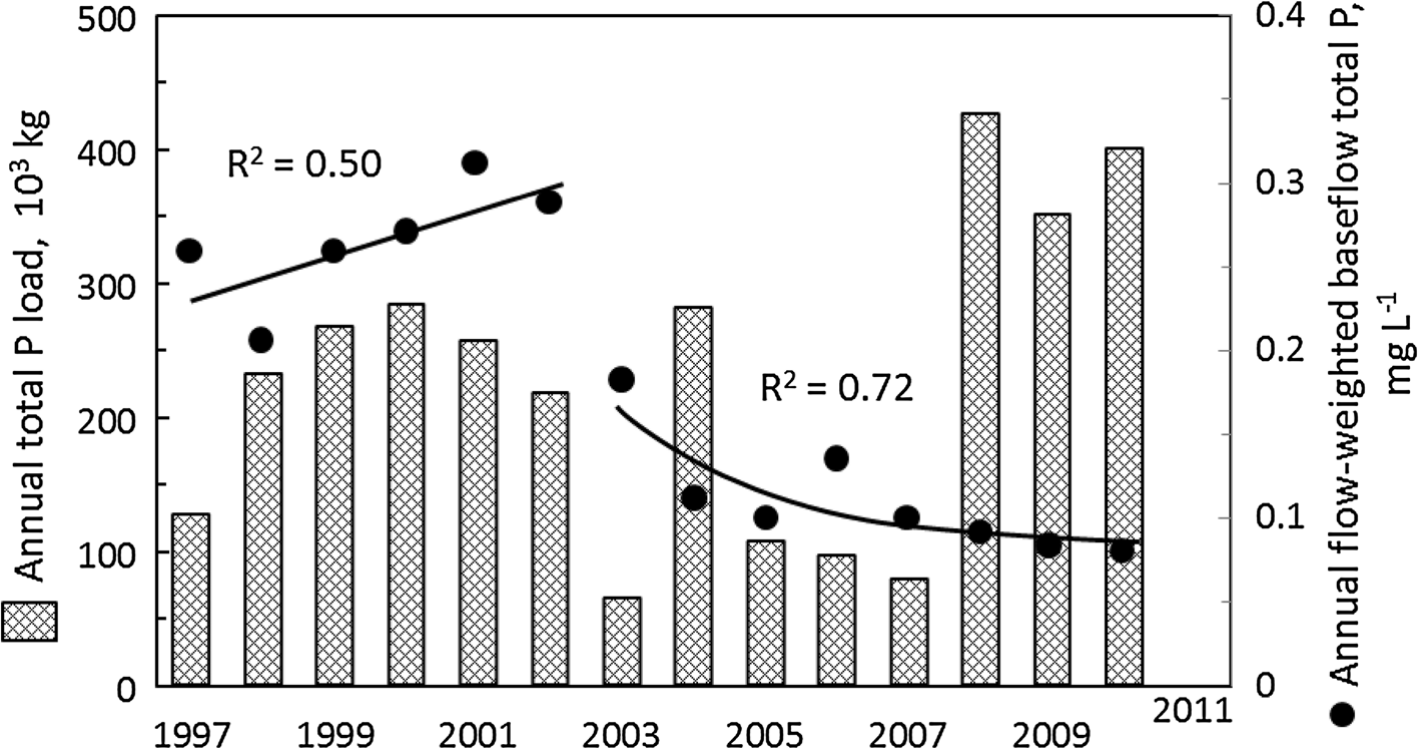
Trends in annual total P loads and mean annual total P concentrations of baseflow in the Illinois River, Arkansas as it flows into Oklahoma at the Highway 59 Bridge USGS sampling site (adapted from Haggard 2010)

In the time following implementation of court-mandated nutrient management changes, there has been a slow but constant decrease in the concentration (mg L^−1^) of total P in baseflow of the Illinois River as it flows from Arkansas into Oklahoma (Fig. 4). Since 2003, required P-risk nutrient management has decreased litter applications to area pastures (Fig. 3) and water treatment plant upgrades have reduced point source inputs of P, making it impossible to isolate the impact of litter export on P loadings to the Illinois River (Haggard 2010). Annual variations in flow have served to hide the effect of lower concentrations (mg L^−1^) of P in baseflow on watershed P losses (kg ha^−1^). However, concentrations have decreased a third compared with pre-2003 (from 0.29 mg L^−1^ in 2002 to 0.07 mg L^−1^ in 2010; Fig. 4), leading to directional trends that provide hope to agricultural and conservation communities alike.

## Voluntary and regulatory experiences in the UK

Phosphorus loadings to freshwaters of the United Kingdom (UK, comprising England, Wales, Scotland, and Northern Ireland) are a major water quality concern; for example in England and Wales, P is the largest single contributor to poor ecological status in rivers and lakes (EA 2013, 2014). Across the UK, under the European Union (EU) Water Framework Directive, targets of annual average concentrations of reactive P in rivers and total P in lakes and reservoirs have been set to help reduce eutrophication and achieve good ecological status (e.g., Ryder and Bennett 2010). Agriculture is a major contributor and is currently targeted for P mitigation (McGonigle et al. 2012; EA 2014; Zhang et al. 2014). However, very different approaches to achieving agricultural P loss controls are used in Great Britain (England, Wales and Scotland), where implementation programs are largely voluntary, versus Northern Ireland, where national regulations are applied across all of agriculture, but have a specific focus on P with regard to farm P budgets and fertilizer use.

### Great Britain’s targeted, voluntary/coercive approach

In Great Britain, specific measures to reduce P pollution have been targeted to ‘sensitive’ watersheds only, using a largely voluntary approach to implementation with knowledge transfer programs and financial incentives to promote adoption (McGonigle et al. 2012). Farmers in Great Britain are bound by the Water Resources Act 1991 (or Scotland’s Water Environment Regulations of 2011) not to cause general water pollution. In addition, under the EU Nitrates Directive to reduce N leaching, farmers face regulatory limits on manure N inputs and closed periods for spreading manures which may also help to reduce P inputs and/or P losses in runoff. To receive EU subsidies, farmers in Great Britain must undertake an annual soil protection review, comply with setbacks, or no spread zones, around watercourses (including ditches) and adopt good nutrient management, all of which are expected to help reduce P loadings to water. For example, farmers are guided not to apply more total P than will be removed by crops in the rotation where soil test P concentrations are already high (Olsen P > 26 mg L^−1^; Defra 2010). However, there exists no specific regulation of P use in agriculture in Great Britain. In effect, the current approach is to wait to see how these general ‘best practice’ measures and gentle coercion under cross-compliance will mitigate watershed P losses before regulatory controls are considered (McGonigle et al. 2012).

To encourage farmers to adopt general water protection and more specific conservation measures to help reduce P delivery to watercourses (e.g., buffer strips, streambank fencing, wetlands), a mix of countryside stewardship programs has been promoted in Great Britain. For example, a major focus on diffuse pollution control based on watershed stakeholder engagement was introduced in 2005 in England and Wales under the Catchment Sensitive Farming Programme (EA 2011). A similar initiative exists in Scotland (McGonigle et al. 2012). Under this programme, knowledge transfer events and targeted visits to farms in sensitive watersheds by government agency staff are designed to improve farmer understanding of the local environment, encourage best practice, implement soil conservation and runoff control measures and address competing priorities. There are positive indicators that the Catchment Sensitive Farming program is improving knowledge, attitudes and decision making related to agricultural impacts on water quality. In England and Wales 80–90 % of the farmers attending watershed extension events felt they were more aware and engaged with diffuse pollution issues, and 50 % of farmers said they would act upon the pollution prevention advice offered by government agency staff (EA 2011). This program has now been extended into the ‘Catchment Based Approach’ which is now the main policy framework for addressing agriculture’s impact on water quality in England and Wales (Defra 2013).

Despite substantial goodwill by the agricultural community toward the Catchment Sensitive Farming Programme, there has been very little evidence that water quality or ecological status in targeted catchments has improved, even though watershed model predictions suggested that the implementation of Catchment Sensitive Farming measures would reduce pollutant loads to water by 5–10 %. Monitoring of selected catchments has suggested small improvements in dissolved and total P concentrations, but it is unclear to what extent these are due to Catchment Sensitive Farming activity (EA 2011; Defra 2014). There is similar experience in Scotland where diffuse pollution mitigation measures have been implemented in watersheds (Bergfur et al. 2012).

### Northern Ireland’s nation-wide, regulatory approach

In contrast with Great Britain’s approach to addressing watershed P mitigation, Northern Ireland has applied a regulatory approach to P management and does not target P based measures to specific watersheds. Previous experience with voluntary programs to lower P inputs to farms was largely unsuccessful and eutrophication is seen as a region-wide problem (Anon. 2003). Since 2006, Northern Ireland has regulated the use of P in agriculture directly through the Northern Ireland Phosphorus (Use in Agriculture) Regulations, and indirectly through the Nitrates Action Programme, introduced in response to the EU Nitrates Directive. These regulations are directed at sources of agricultural P across the territory.

**Fig. 5:**
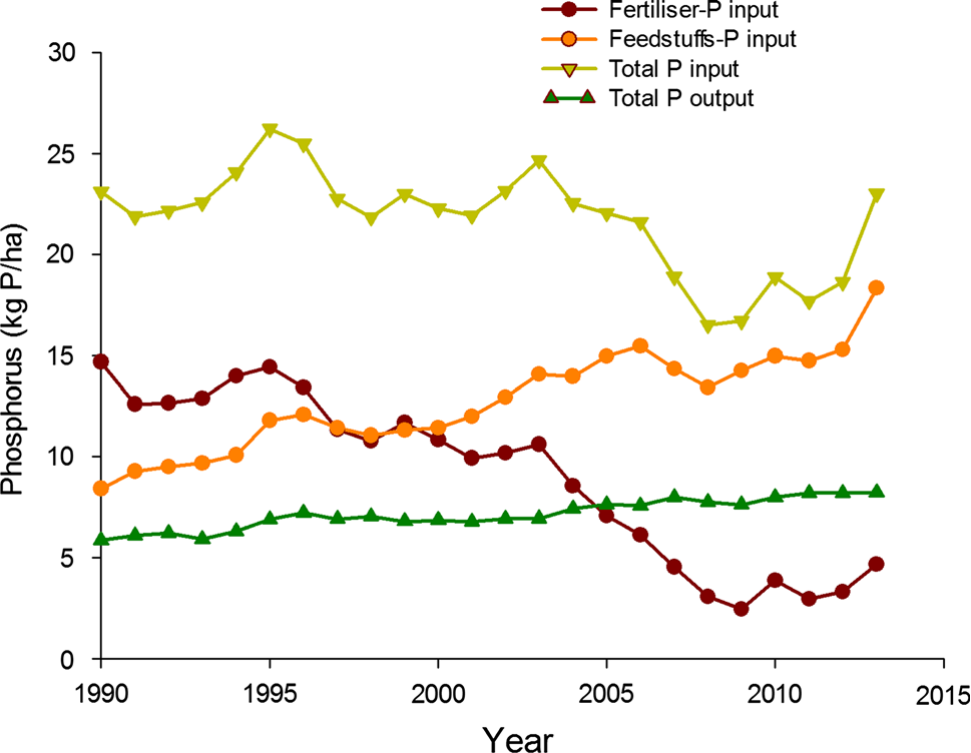
Inputs of fertilizers and feeds into Northern Ireland agriculture between 1999 and 2011 in relation to total outputs of P

The introduction of P regulations in 2006 restricted the application of commercial P fertilizer using a national fertilizer manual (Defra 2010) but did not explicitly restrict manure application on a P basis (as is recommended in Great Britain). Instead, manure application was regulated on an N basis (170 kg N ha^−1^) and for periods of time set by the EU Nitrates Directive. Since 2006, the national P surplus has declined from 14 to 9.5 kg P ha^−1^ in 2011 (Fig. 5). It is unclear how important the Northern Ireland regulations were to this trend: a voluntary agreement with the animal feed industry lowered the national average P content of animal feed from 0.59 to 0.51 %; and, an increase in fertilizer costs likely contributed to lower P fertilizer demand (from 6.1 kg P ha^−1^ in 2006 to 3.3 kg P ha^−1^ in 2011). Indeed, in 2013, P fertilizer use increased to 4.7 kg P ha^−1^, helping to push the national surplus back up to 12.3 kg P ha^−1^ year^−1^ (Fig. 5). The reason for this increase is unclear. Despite uncertainties in causality, the ability to distribute credit for long-term declines in agricultural P use has increased cooperation between regulators and the agricultural industry alike.

Northern Ireland’s non-point source regulations have yielded similarly uncertain outcomes as those documented under Great Britain’s voluntary programs. Half of all rivers in Northern Ireland remain classified as “moderate/poor status” under the Water Framework Directive and 70 % of lakes are still classed as eutrophic. In recent years the Northern Ireland monitoring program has observed that, following initial decreases in dissolved P concentrations in many rivers, concentrations have been stabilizing at levels above the threshold values required to achieve “good ecological status” under the Water Framework Directive.

### Confronting P-based management and monitoring in the UK

Despite some indicators of success, neither the voluntary/coercive approach to mitigating agricultural P loss in Great Britain, nor the more regulatory approach to improving farm-gate P budgets and fertilizer management adopted in Northern Ireland, have shown sufficient improvements in water quality to suggest any of these approaches can fully address the non-point source pollution risks contributing to eutrophication in the UK. There are no shortage of hypotheses and accusations, especially when the non-point source pollution programs are held up to the water quality successes of point source programs in the UK and indeed across Europe (Bowes et al. 2011; Vaughan and Ormerod 2012; Miller et al. 2014).

One strong likelihood in the UK is that the management programs are insufficiently focused on the specific practices or general strategies required to comprehensively address P loss from agriculture. Another is the reluctance to compromise agricultural productivity and its necessary expansion, especially in areas such as Northern Ireland where it may not be possible to farm intensively and protect water quality (Doody et al. 2014; Withers et al. 2014). The causes of these shortcomings and valid productivity concerns are manifold, but must be considered. In Great Britain limited adoption is clearly a first tier concern, but then there are the specifics of which practices are implemented. Currently, there is a hope that practices improving nutrient use efficiency or broadly applicable to soil conservation and nutrient management will be sufficient. In Northern Ireland, reliance on reducing P surpluses to control P loss, principally through focus on commercial fertilizers, may not be sufficient in many watersheds due to local factors having a greater influence on P transfer from land to water than the size of the surplus: manure management; soil P binding capacities; variable losses of legacy P; hydrological connectivity (Jordan et al. 2005; Cherry et al. 2008; Jordan et al. 2012; Sharpley et al. 2013).

In the livestock industry in the UK, the theme of legacy P (P that resides in the soil and sediments from historical applications of fertilizer and manure) is one of the most difficult subjects to broach. Deep seeded opinions exist over the use of soil testing to regulate manure application which would have a major impact on available land areas for spreading. As agriculture in Northern Ireland is predominately grassland based and up to 50 % of soils in intensively farmed areas have P levels above the agronomic optimum concentrations (16–25 mg L^−1^ Olsen P), restricting manure application on the basis of soil P would severely limit the recycling of livestock manures and adversely impact on farm businesses. Similar concerns exist in areas of Great Britain where high livestock densities have led to high soil P levels. In this respect the focus on P surplus reduction in Northern Ireland as a remedy for eutrophication control is a sacrificial lamb that ignores the role of legacy P and hydrological connectivity in watershed P export. The lack of comprehensive soil testing programs to inform P management at field, watershed and regional levels means that legacy soil P remains a significant threat to water quality in many UK watersheds (Doody et al. 2012; Jarvie et al. 2013; Withers et al. 2014).

Clear opportunity exists to refine water quality monitoring and goals to more accurately identify sources of watershed P loads and to set appropriate expectations. Indeed, P concentration targets for eutrophication control in UK freshwaters are very challenging in relation to current demography (ca. 250 capita km^2^), and intensity of land use (over 70 % of managed land). The ecological relevance of agricultural P loadings to eutrophication risk in rivers is currently not considered and there remains much debate over whether the eutrophication impact of agricultural sources is over-estimated for many river catchments in the UK when assessed on the basis of their contribution to total annual load (Withers et al. 2014). In a recent analysis of 15 tributary sub-watersheds of the River Thames, agriculture was found to be the dominant source of P in all cases based on its contribution to annual loadings of total P (Bowes et al. 2014). Loadings of P to these tributaries during the ecologically active period were, however, dominated by wastewater discharges in all but three cases. A move toward more regime-based P targets, based on waterbodies sensitivities to the bioavailability, timing and mode of P inputs, maybe more successful in achieving more rapid and lasting water quality improvements (Page et al. 2010; Jarvie et al. 2013).

## Sweden’s actions under the Baltic Sea Action Plan

Of the case studies reviewed here, Sweden represents the most tightly regulated setting for agricultural P-based management, with a great portion of the costs related to P mitigation measures covered by subsidies. Agricultural P management in Sweden coupled to eutrophication of the Baltic Sea is today, to a large extent, driven by the Baltic Sea Action Plan (BSAP), an international accord that was devised in 2007 after P was implicated as the main cause of cyanobacterial blooms in the Baltic Sea (Boesch et al. 2006). Despite generally low intensity of land use, Swedish agriculture is estimated to account for 40–50 % of the total anthropogenic P loads from the nation’s Baltic watersheds (Brandt et al. 2009). To meet the targets prescribed in BSAP and achieve the P load reductions required, national regulations related to e.g., spreading of animal manure (limited to 110 kg P ha^−1^ over a 5-year period) and the EU Water Framework directive need to be followed. In addition, a voluntary advisory program (‘Greppa näringen’), which was introduced in 2000 to give farmers in sensitive areas free individual support, has helped to reduce agricultural P losses at a cost of about $4 million USD year^−1^.

A central theme of Swedish P mitigation programs is to pay farmers for conservation and nutrient management measures they adopt. Since 2000, subsidies have been available through the Swedish Rural Development Program, which is partly funded by the EU, to compensate farmers for carrying out certain practices to reduce both N and P losses. Practices qualifying for such subsidies especially related to P include conservation buffer zones for highly erodible soils, constructed wetlands, and, perhaps most notably, organic crop production, which is a subsidy to help reduce environmental disturbances in general. In recent years additional mitigation strategies have been included, such as the installation of drainage management practices on tile drains and ditches, i.e., ‘controlled drainage’. Subsidy of water quality mitigation practices has helped to spur adoption of practices aimed at preventing P losses from agriculture, but has not removed some of the profound obstacles encountered under less-regulated, less-subsidized settings.

Although many practices promoted for water quality protection have widespread support in the agricultural community, artificial drainage represents as much of a sacred cow in Sweden as it does in Lake Erie. About 50 % of arable land in Sweden is tile drained, especially those soils with high clay content. As with Lake Erie, artificial drainage is imperative to allow field management operations to be performed as early as possible in spring and to protect crops from flooding. However, because artificial drainage is seen as such an essential part of agricultural infrastructure, few in the agricultural community have to date been willing to open discussion on options for limiting new drainage or removing drainage to control non-point source pollution. Instead, other measures to improve soil structure such as liming of clay soils and tile-drainage backfills to increase P adsorption to the soil matrix have been tested with good results (Ulén and Etana 2014). Also, grass buffers along rivers and open ditches have been emphasized and perhaps even over-implemented in Sweden, especially when viewed through the lens of P mitigation. Grass buffers are used as a multifunctional tool in agricultural landscapes around the world, providing many ecosystem services other than the regulation of nutrients and sediments (Stutter et al. 2012). For P, buffer zones are thought to be effective in promoting sedimentation and retention of sediment-bound P, but their efficacy in preventing dissolved P loss has been widely questioned (Dorioz et al. 2006). Indeed, when buffer zones are bypassed with concentrated flow pathways or when the P-binding capacity of the soils is largely saturated, they can range from ineffective to a source of P (Uusi-Kämppä and Jauhiainen 2010). Even so, from 2000 to 2006, Swedish farmers received about $3 million USD year^−1^ in subsidies to install and maintain riparian buffer zones, with an estimated reduction in watershed P loads of 6 tons year^−1^, equivalent to a total P removal efficiency of $500 kg^−1^ year^−1^.

National debate over the cost effectiveness of buffer zones as P mitigation tool is largely stifled by the amount spent on agricultural subsidies in Sweden for organic agriculture, effectively turning buffer zones (or any other P mitigation efforts) into a sacred cow for Swedish taxpayers. Recently, an evaluation of the Swedish Rural Development Program concluded that programs to reduce agricultural non-point source pollution with specific practices such as buffer zones were much more cost-effective than national subsidies for organic crop production, for which Swedish farmers received $75 million USD year^−1^ from 2000 to 2006. In fact, it can be argued that subsidies for P mitigation practices, regardless of their cost effectiveness, help to offset the P losses from organic farms, which are sometimes greater than from conventional systems (Aronsson et al. 2007).

When Sweden’s P mitigation programs are evaluated on the basis of water quality alone, without considering cost, there is cause for cautious optimism. Downward trends in P concentrations in large rivers in agricultural areas of southwest Sweden have been noticed, although, the picture is somewhat diverse with increasing trends in some rivers (Fölster et al. 2012). Thus, implementation of mitigation strategies in Sweden has had a slight beneficial effect on reducing agricultural P losses, but it is too early to draw any general conclusions.

## Conclusions

Recent experience with the implementation of P mitigation strategies under regulatory and volunteer settings points to a core set of issues confronting success. Dissolved P losses from agriculture appear to be particularly difficult to control. Uncertainty over the outcome of implementation strategies, an inherent aspect of diffuse P pollution creates room for competing perspectives on the best practices to mitigate P loss and the most appropriate means for implementing these practices. Unintended consequences of past management efforts or current initiatives appear to be the rule, rather than the exception, from conflicts between soil conservation and dissolved P management to adverse impacts on producers who have benefited from cheap, local sources of livestock manure. Since P-based management often requires activities that are separate from standard practice and incur additional costs, subsidies are a natural outcome of government-led mitigation efforts. These subsidies can be important to the success of programs, but they are by no means a universal requirement for success. In all cases, local empirical data, particularly in the form of water quality monitoring, are the best means of convincing skeptics and compelling local responsibility to act. Indeed, without adequate local information on practice adoption and effectiveness, whether imposed by regulation or adopted on a voluntary basis, it is impossible to judge the success and failure of mitigation programs.

The commonalities observed between case studies should not obfuscate the imperative for local, site-specific consideration of practice potential, compatibility and effectiveness. In the Western Lake Erie, a relatively recent reversal of historical water quality improvement recently culminated in the temporary closure of public drinking water supplies for the City of Toledo in 2014. This event has elevated concerns even higher and has created a politically charged environment in which ready accusations from certain sectors have placed a heavy onus on waiting for locally derived empirical findings. In this environment, uncertainty and competing interests within the farm and conservation community have introduced certain practices (especially those involving tillage), and, more importantly, unnecessary conflict.

In the Illinois River and Eucha–Spavinaw watersheds, USA, litigation amplified differences between stakeholders in the states of Arkansas and Oklahoma. Most notably, regulations derived from the lawsuit were initially feared as sufficiently draconian to drive away many poultry farmers. However, a successful litter export program and subsidies were key to success and to minimizing adverse impacts to poultry farmers. An unanticipated casualty of the P-based regulations, however, has been the beef farmers, who have been dependent upon local sources of poultry litter to improve pasture conditions and sustain beef herds.

In the UK and Sweden, multinational EC and Baltic State agreements set a framework in which P-based management strategies have been promulgated. These strategies differ widely across partner states, even within the UK. In some cases, the implementation of management programs has coincided with separate, industry-led initiatives, resulting in positive outcomes that are not necessarily the product of the government program. For most programs, the key metric of success for most management programs is the extent to which a practice is implemented, rather than the effectiveness of practice adoption in mitigating water quality degradation. Better ties between practice implementation and water quality benefit are required to ensure cost-effective implementation programs.
